# A topological proof of the modified Euler characteristic based on the orbifold concept

**DOI:** 10.1107/S2053273321004320

**Published:** 2021-06-21

**Authors:** Bartosz Naskręcki, Zbigniew Dauter, Mariusz Jaskolski

**Affiliations:** a Faculty of Mathematics and Computer Science, A. Mickiewicz University, Poznań, Poland; b Macromolecular Crystallography Laboratory, NCI, Argonne National Laboratory, Argonne, Illinois, USA; c Department of Crystallography, Faculty of Chemistry, A. Mickiewicz University, Poznań, Poland; d Center for Biocrystallographic Research, Institute of Bioorganic Chemistry, Polish Academy of Sciences, Poznań, Poland

**Keywords:** Euler characteristic, orbifolds, space-filling polyhedra, space groups, asymmetric units

## Abstract

The notion of the Euler characteristic of a polyhedron or tessellation has been the subject of in-depth investigations by many authors. Two previous papers worked to explain the phenomenon of the vanishing (or zeroing) of the modified Euler characteristic of a polyhedron that underlies a periodic tessellation of a space under a crystallographic space group. The present paper formally expresses this phenomenon as a theorem about the vanishing of the Euler characteristic of certain topological spaces called topological orbifolds. In this new approach, it is explained that the theorem in question follows from the fundamental properties of the orbifold Euler characteristic. As a side effect of these considerations, a theorem due to Coxeter about the vanishing Euler characteristic of a honeycomb tessellation is re-proved in a context which frees the calculations from the assumptions made by Coxeter in his proof. The abstract mathematical concepts are visualized with down-to-earth examples motivated by concrete situations illustrating wallpaper and 3D crystallographic space groups. In a way analogous to the application of the classic Euler equation to completely bounded solids, the formula proven in this paper is applicable to such important crystallographic objects as asymmetric units and Dirichlet domains.

## Abstract 2

The vanishing of the modified Euler characteristic for symmetrically arranged space-filling polytopes is given a general proof based on the topological concept of orbifolds. The modified Euler characteristic is applicable to such important crystallographic objects as asymmetric units and Dirichlet domains.

## 1. Introduction   

The famous Euler formula *V* − *E* + *F* = 2 applies to any single 3D solid (polytope) with *V* vertices (or 0-cells), *E* edges (1-cells) and *F* faces (2-cells) that is completely bounded by those ‘surface’ elements. It could be a crystal, its model or indeed any isolated solid, for example an icosadeltahedron representing a spherical virus particle. However, if the solid is not completely bounded, as is the case with space-filling polytopes such as the crystallographic asymmetric units which share the bounding elements with their neighbors, the Euler sum is reduced by one, and the analogous modified (m) formula takes the following form.

Theorem 1.1 The modified Euler characteristic of a space-filling polytope 

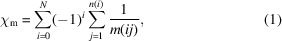

where we count each *j*-cell with a weight inversely proportional to its multiplicity *m*(*ij*) and up to dimension *N*, satisfies the equality 

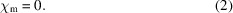

The way to prove this theorem is to reinterpret first the formula χ_m_ as the orbifold Euler characteristic 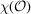 (Section 3) of the orbifold 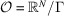 (Definition 7.2[Statement definition7.2] and Theorem 7.1[Statement theorem7.1]) for the space group Γ acting properly discontinuously (Definition 7.1[Statement definition7.1]) on the Euclidean space 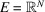 , and then to prove 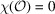 in Theorem 4.2[Statement theorem4.2].

The property χ_m_ = 0 proved above, in the form *V*_m_ − *E*_m_ + *F*_m_ − 1 = 0, was noted by Dauter & Jaskolski (2020 ▸) and demonstrated for all standard 2D and 3D asymmetric units as well as for Dirichlet domains. In a related paper (Naskręcki *et al.*, 2021 ▸) this property is expressed using the topological notion of the Euler characteristic [which is an alternating sum of all *i*-cells from *i* = 0 to *N*: 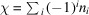 ] with the inclusion in the summation of the polytope itself (*I* or ‘interior’, or *N*-cell in 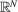 ). In the paper by Naskręcki *et al.* (2021 ▸) a rigorous proof of the vanishing of the modified Euler characteristic is also presented using a simple property of parity group frequencies, but [like a similar but much more tedious proof outlined by Coxeter (1948 ▸), equation 4.82] it is strictly applicable to translational tessellation of space (*i.e.* to the unit cell) and has to be extended to the asymmetric unit (ASU) using the theorem presented by Whitehead (1949 ▸) and Hatcher (2002 ▸). In the present paper, we provide a completely general proof (applicable to any periodic tessellation of space of any dimension by identical polytopes) rooted entirely in the topological notion of the orbifold and its properties.

Our aim in Section 2 is to provide a topological explanation for the phenomenon of the vanishing of the modified Euler characteristic as observed by Dauter & Jaskolski (2020 ▸). We model our approach on the theory of orbifolds as introduced by Satake (1956 ▸) (under the name of *V*-manifolds) and Thurston (2002 ▸).

First, we introduce the necessary topological notions of manifolds and orbifolds. Next, we discuss the essential properties of groups acting on topological spaces, and then we discuss how the Euclidean 3D space *E* with the action of the space group Γ can be considered in this new setting.

Our goal in Section 3 is to demonstrate the vanishing of the Euler characteristic from the very basic topological properties of the orbifolds attached to the pair (*E*, Γ). We recall the fundamental properties of the Euler characteristic, which are well known to topologists, *i.e.* invariance under the change in cell decomposition, and the multiplicativity property for finite covers of spaces. These ideas are extensively discussed in the excellent textbook by Thurston (2002 ▸). We intertwine the abstract topological concepts with down-to-earth examples to illustrate our concepts.

In Section 4, we present the proof of Theorem 4.2[Statement theorem4.2] which explains why the modified Euler characteristic attached to the pair (*E*, Γ) vanishes in every case. The proof is brief and uniform, without going into detailed combinatorics of particular examples, and our argument applies to the action of crystallographic groups in arbitrary dimension.

## 2. Manifolds and orbifolds   

An orbifold is a topological notion similar to a manifold. While manifolds are simply ‘pieces’ of the Euclidean space 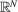 glued together by continuous maps, the former are more complicated to describe. To state it simply, we will mainly discuss manifolds and orbifolds which are subsets or quotients of the Euclidean space 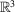 . A manifold consists of a space *X* with an atlas of charts {(*U*, ϕ)} which satisfy certain compatibility conditions [see Lee (2003 ▸) and Millman & Parker (1977 ▸)]. On a manifold *M*, a chart (*U*, ϕ) is a pair of an open subset 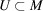 and a homeomorphism 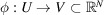 where *V* is an open subset. An atlas is a collection {*U*_*i*_, ϕ_*i*_}_*i*∈*I*_ of charts indexed by some set *I* and such that 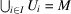 .

For example, a 2D sphere 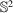 is a subset of 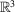 that consists of triples of real numbers (*x*, *y*, *z*) which satisfy the relation *x* ^2^ + *y* ^2^ + *z* ^2^ = 1. As a manifold, it can be characterized by the following atlas. We consider 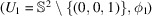 to be the stereographic projection of ϕ_1_ from the ‘north pole’ at (0, 0, 1) onto the plane *z* = −1. Its inverse 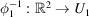 is defined as 

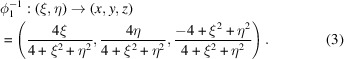

This map is a continuous bijection which covers all but one point on the sphere. Similarly, we have a projection from the ‘south pole’ at (0, 0, −1) which provides the second chart 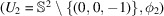 with analogous formulas. The overlap *U* of the two charts is the sphere 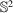 with two points removed, (0, 0, 1) and (0, 0, −1), and with a composite map 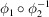 which is a continuous bijection on 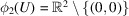 . The role of the atlas is to provide a ‘navigation’ on the manifold under consideration. Once given, one can completely forget about the extrinsic model of 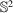 and describe the whole topology using only the charts.

An orbifold is a similar notion where the open sets 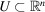 are replaced with *U*/*H*, where *H* is a finite group and *U*/*H* is an orbit space (Satake, 1956 ▸; Thurston, 2002 ▸). We also point to the work of Conway *et al.* (2008 ▸) for an intuitive introduction to orbifolds.

The underlying space of the orbifold is usually not as nice as for the manifold. It typically contains points which have a neighborhood that does not resemble the usual Euclidean space (singular points). This is caused by the application of the group quotient. For the necessary definitions of the group actions, quotients, orbifold atlas and orbifold covers that will be used later, we refer the reader to Definitions 7.1[Statement definition7.1], 7.2[Statement definition7.2] and 7.3[Statement definition7.3] in Appendix *A* [App appa].

In a given orbifold 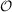 we distinguish the set 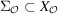 of singular points. Each point 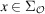 has the property that in the chart 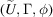 where 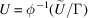 is a neighborhood of *x*, any pre-image 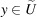 of ϕ(*x*) has a non-trivial stabilizer Γ_*y*_ ≠ 0. All these groups are conjugate and we denote any representative of the conjugacy class by Γ_*x*_ and call it the local group of *x*. All points 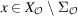 are called non-singular and their local group Γ_*x*_ is trivial.

For each singular point *x* of an orbifold space 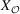 of a given orbifold 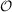 , we consider a maximal connected set *Z*_*x*_ in 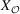 of points 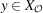 for which the local groups Γ_*y*_ are the same up to conjugation. This leads to a natural stratification of the space 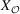 into a union of relatively closed connected submanifolds, where on each stratum the local group (up to conjugation) is constant for each point in the interior. Such a stratification is called the local group stratification.

### 2.1. Example of an orbifold   

**Figure 1:**
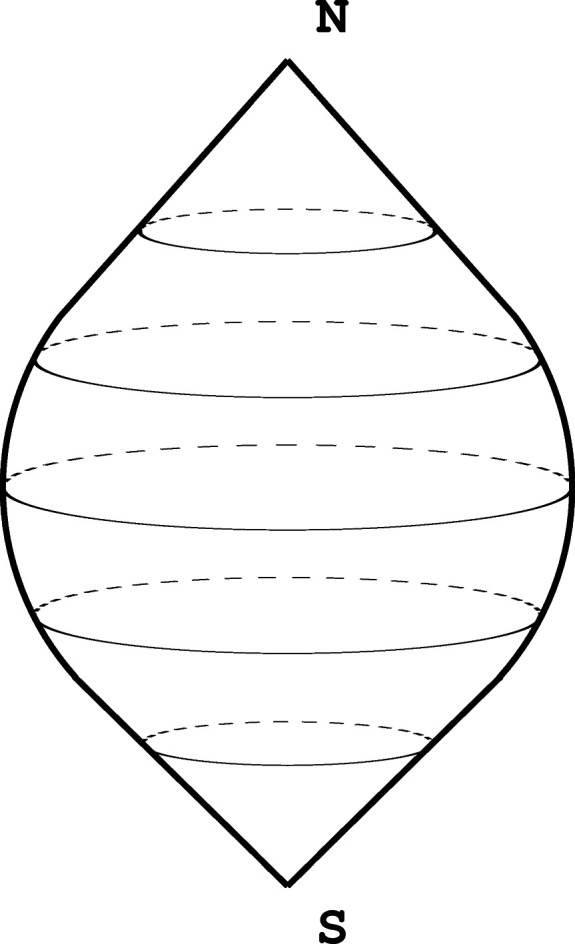
An illustration of the 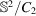 orbifold with two singular points.

The rotation of 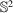 around the *z* axis by 180° provides a group action of the cyclic group *C*_2_ of order 2 on the space 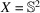 . This action has two obvious fixed points at the poles (0, 0, 1) and (0, 0, −1) and nowhere else. The quotient space *X*/*C*_2_ (Fig. 1 ▸) is topologically identified with the sphere itself, but the neighborhoods of the two poles are isomorphic to the orbit space 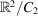 with the action of *C*_2_ by rotations. The stabilizer group of both poles is *C*_2_ and, since the action is properly discontinuous, this provides a way of introducing an orbifold atlas on *X*/*C*_2_. The natural projection *p* : *X* → *X*/*C*_2_ is an example of an orbifold cover, where the structure of the orbifold on *X* is trivial (each point has a trivial stabilizer) and the degree of this cover *p* equals 2, the order of the group *C*_2_. The pre-images of the points under the map *p* consist of two-point sets, except for the pre-images of the points with a nontrivial stabilizer, where they consist of a single point. More examples can be found in the work of Caramello (2019 ▸), Thurston (2002 ▸), Choi (2012 ▸), Conway *et al.* (2001 ▸) and Conway & Huson (2002 ▸).

## 3. Orbifolds associated with space-filling polyhedra   

Since each crystallographic space group Γ acting on the Euclidean space 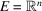 is acting properly discontinuously, the quotient space of orbits *E*/Γ has a natural structure of an orbifold (Theorem 7.1[Statement theorem7.1]). We denote by Isom(*E*) the group of isometries of *E* and by Trans(*E*) the subgroup of translations (Farkas, 1981 ▸).

Definition 3.1: index of a subgroup Let 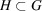 be a pair of groups. The set *G*/*H* = {*gH* : *g* ∈ *G*} is the set of cosets *gH* for every *g*. If the set *G*/*H* is finite, we denote its cardinality by [*G* : *H*] and call it the index of the group *H* in *G*.

Definition 3.2 A free abelian group is a group isomorphic to a product of copies of the unique infinite cyclic group 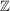 of integers with addition.

It follows from the Bieberbach theorem [Farkas (1981 ▸), Theorem 14] that a crystallographic subgroup Γ of Isom(*E*) has a finite-index free abelian subgroup Λ = Γ ∩ Trans(*E*) of rank dim *E*.

The quotient space *E*/Λ is a 3D torus. The point group *G* = Γ/Λ is finite and determines a finite cover 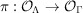 of orbifolds 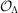 , 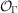 of degree |*G*|.

An important point to make is that every manifold and orbifold that we study in this paper has a structure of a CW complex (cellular complex; Hatcher, 2002 ▸) or in particular admits a triangulation of the space. A triangulation is a structure on the space *X* which allows one to glue it from ‘topological triangles’, *i.e.* continuous images of the *k*-dimensional simplices 

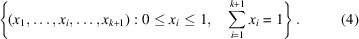

Each *k*-simplex can be glued to another *k*-simplex only along a (*k* − 1)-dimensional simplex, *etc.*

**Figure 2:**
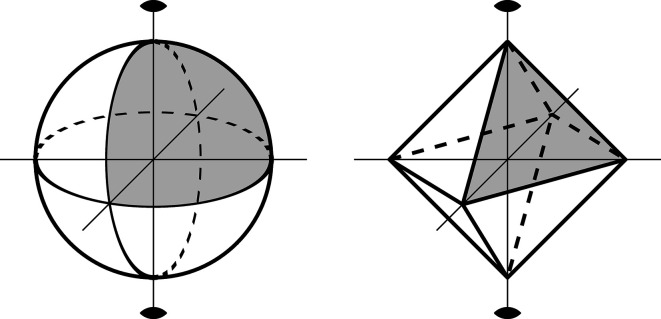
Cellular division of a sphere and an octahedron.

For example, a sphere 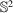 can be triangulated in the following way. We declare six 0-simplices which correspond to the points (0, 0, ±1), (0, ±1, 0), (±1, 0, 0). These are glued together by images of twelve 1-simplices that correspond to pieces of large circles connecting each pair of 0-vertices contained in the same hemisphere. The regions into which the sphere is divided by the 1-simplices are images of 2-simplices and there are eight of them. Note that this structure corresponds to the division of the octahedron into vertices, edges and faces (Fig. 2 ▸).

A more compact way of describing a space is when, instead of dividing it into simplices, we divide it with respect to a cellular division. The building blocks in this case are *n*-balls (called *n*-cells) which are glued along their boundaries in a much more flexible way than the structure of the simplicial complex (Hatcher, 2002 ▸). For the sphere 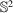 one takes a closed disk *B* ^2^ and glues it along its boundary to a single point. Topologically, this corresponds to the 1-point compactification of the plane obtained by stereographic projection from one point on the sphere.

Every orbifold obtained from the quotient of the Euclidean space by a crystallographic group has a triangulation which is compatible with the local group stratification (Choi, 2012 ▸). It means that one can introduce a cell division on the underlying space *X* of the orbifold such that for each cell there exists a group Γ such that for each point *x* that belongs to the interior of that cell the stabilizer group Γ_*x*_ equals Γ.

For the example of the *C*_2_ action on the sphere 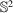 , the quotient space is divided into cells as follows: two 0-cells which correspond to the south and north poles (0, 0, ±1). These two points have the stabilizer group equal to *C*_2_. The *C*_2_ action wraps the large circle going through the poles onto itself, so that it becomes a half circle with endpoints at the poles. The points in the interior (*i.e.* away from the endpoints) of this half circle have a trivial stabilizer (they have two points in the pre-image of the projection map 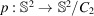 ). The large circle described above is a boundary of two hemispheres which map under the *C*_2_ action onto each other. One of these hemispheres is taken as the unique 2-cell in this cellular decomposition and the points in the interior are not fixed by *C*_2_ again.

## 4. Proof of the main theorem   

In this section, we finally provide the proof of our main theorem. Thurston (2002 ▸) generalized the notion of the Euler characteristic of a manifold to a setting of orbifolds. Following his work we define it in the following way.

Definition 4.1: orbifold Euler characteristic For an orbifold 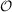 and its underlying space 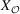 , the Euler characteristic 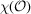 is defined as 

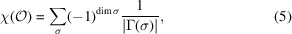

where the summation goes over cells σ of the cell division of the space 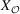 and Γ(σ) is the stabilizer group of cell σ. The cell division must respect the constancy of the stabilizer group Γ_*x*_ of each point *x* in the interior of each cell σ. We denote by 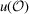 the vector 

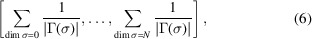

where *N* is the top dimension of the cell in 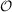 . We call 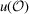 the weighted *k*-cells vector of the orbifold 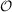 .

In contrast to 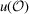 , the definition of 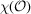 does not depend on the particular choice of cell division and is always a finite number when the orbifold is compact (which is the case for 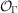 and 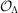 ). This is a consequence of the homological interpretation of the Euler characteristic [Hatcher (2002 ▸), Theorem 2.44]. The orbifold Euler characteristic 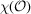 of the orbifold 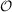 which is modeled on a crystallographic ASU provides a very elegant and formal interpretation of the modified Euler characteristic χ_m_ introduced at the beginning.

Example 4.1 The orbifold Euler characteristic of a 3D torus 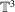 equals 0. We consider the torus 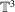 with a trivial group action. We are going to justify this below.

We consider the space 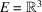 and the group of unit translations 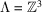 acting freely on *E*. The fundamental region of this action is a unit cell without appropriate faces. The underlying space of the orbifold *E*/Λ is the torus 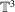 . To compute its Euler characteristic, we consider the cell decomposition in which we have one 0-cell, which corresponds to the point *V* = (0, 0, 0). We have three 1-cells *E*_*x*_, *E*_*y*_, *E*_*z*_ that correspond to three unit segments that stem from *V*. The 1-cells *E*_*x*_, *E*_*y*_, *E*_*z*_ span, pairwise, three faces (2-cells) *F*_*x*_, *F*_*y*_, *F*_*z*_ and we have a unique 3-cell *I*. The stabilizer group Γ(σ) is trivial for each cell since the group action Λ is free on *E*. Hence, 

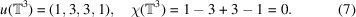

This of course agrees with the Euler characteristic of 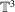 computed in the standard way, as well as with the Euler number computed as the sum 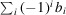 of the topological Betti numbers *b*_*i*_ [Hatcher (2002 ▸), Theorem 2.44 and Example 2.39].

A similar calculation leads to the conclusion that an *N*-dimensional torus has the Euler characteristic equal to 0. An elegant arithmetical interpretation of this fact is presented by Naskręcki *et al.* (2021 ▸).

Theorem 4.1 [Thurston (2002), Proposition 13.3.4; Choi (2012), Proposition 5.1.3] For a finite cover of degree *d* of orbifolds 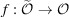 it follows that 

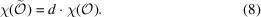

Example 4.2 For the sphere 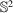 and its degree 2 orbifold cover 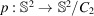 we compute the Euler characteristic. The Euler characteristic of 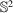 equals 2, which follows from the octahedral triangulation of the sphere. The cell decomposition of the orbifold 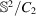 contains two 0-cells with stabilizer group *C*_2_, one 1-cell with a trivial stabilizer and one 2-cell with a trivial stabilizer: 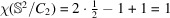 . The degree of the cover *p* is indeed 2 and hence 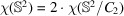 .

Proof of Theorem 4.1 The proof of (8) follows from the simple group theoretic properties of the orbifold covers [Thurston (2002 ▸), Proposition 13.3.4; Choi (2012 ▸), Proposition 5.1.3]. The degree *d* of the cover *f* is equal to 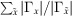 , where the sum runs over 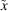 points which map to *x, i.e.* 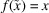 and *x* is an arbitrary point of 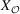 . Note that the number *d* equals the number of pre-images in the set *f* ^−1^(*x*) if the stabilizer groups Γ_*x*_, 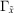 are trivial. If they are not, this follows from the study of the number of points in the pre-image of a regular point *y* in the neighborhood of *x*. Alternatively, we can look at the pre-images of cells under the covering map.

To prove the theorem, it is enough to note that any point 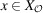 belongs to the interior of a unique cell in the cell decomposition of 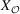 . Thus, the stabilizer group Γ_*x*_ is actually the stabilizer group Γ(σ) of the cell σ such that *x* ∈ σ. For a point *x* ∈ σ in the interior of σ, we have 

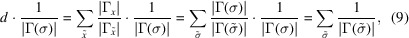

where the summation over 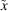 is such that 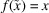 and the summation over 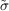 is over cells 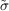 such that 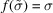 . Taking the sum over all cells σ we obtain 

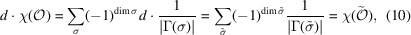

which finishes the proof. Note that the equality 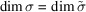 holds for any 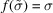 since the cover *f* has a finite degree.

□

Theorem 4.2 The orbifold 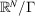 obtained from the action of the crystallographic space group Γ on the Euclidean space 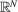 has an orbifold Euler characteristic equal to 0.

Finally, we are ready to prove that for an ASU 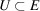 , the modified Euler characteristic of *U* computed by Dauter & Jaskolski (2020 ▸) must be equal to 0. This is equivalent to proving that the orbifold Euler characteristic of the space 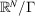 for a space group Γ vanishes.

Proof of Theorem 4.2 We impose a cell structure {σ} on the region *U*, which propagates via the crystallographic space group Γ to the cell structure on the Euclidean space *E*. Our choice of the cell structure is compatible with the action of group Γ, *i.e.* for every cell 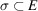 , a copy γ · σ generated by the element γ ∈ Γ is again a cell in the decomposition of *E*. Note that the number of cells which impose a cell decomposition on *U* is finite, *i.e.* 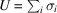 for finitely many cells σ_*i*_. By the symmetry reconstruction process, we obtain the decomposition 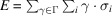 where the intersections γ · σ_*i*_ ∩ γ′ · σ_*j*_ are unions of cells of the same decomposition.

The orbifold 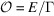 has an underlying space 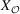 which is obtained by appropriately glueing and identifying the cells in the decomposition of *E*. That imposes a natural cell decomposition 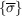 of 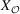 where each cell 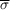 has infinitely many cells σ which map onto it by the projection map 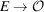 . These cells in the pre-image lie in the same orbit with respect to the group action of Γ. The stabilizer group 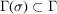 of cell σ is finite.

As mentioned above, it follows from the Bieberbach theorem [Farkas (1981 ▸), Theorem 14] that there is a finite-index subgroup Λ isomorphic to 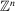 contained in Γ. The inclusion 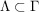 induces an orbifold cover 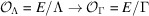 of degree [Γ : Λ]. It follows from Example 4.1[Statement example4.1] and (8) that the orbifold Euler characteristic of 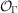 vanishes.

□

### 4.1. Examples of orbifolds which arise from crystallographic space groups   

#### 4.1.1. The wallpaper group *p*3   

**Figure 3:**
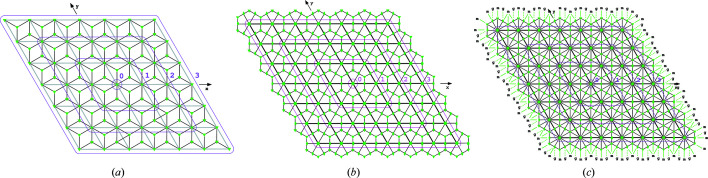
Orbifold ASU tessellation of the groups (*a*) *p*3, (*b*) *p*6 and (*c*) *p*6*mm*. A purple rhombus of index *i* represents a region which encircles the elements of the tessellation which are subject to a counting function with parameter *i*.

In this symmetry the ASU is given by a rhombus, the vertices of which are stabilized by the symmetries of order 3. The orbifold 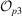 associated with this construction has three vertices of weight 1/3, two edges with endpoints at these three points, both of weight 1, and finally a single face of weight 1. The details of this construction are contained in Figs. 3 ▸(*a*) and 4 ▸. The total vector of weighted *k*-cells is 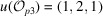 and 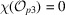 .

**Figure 4:**
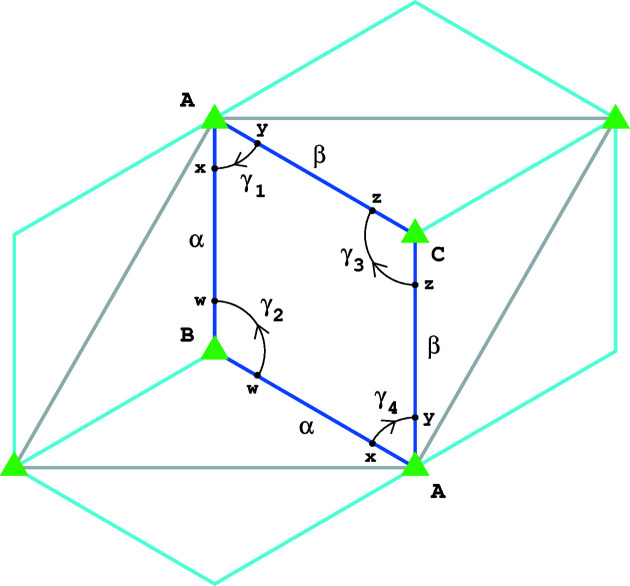
The unit cell (gray edges) and the ASU (blue edges) with four adjacent copies of the ASU (light blue) of the wallpaper group *p*3. The green triangles indicate points with rotational threefold symmetry. The arcs indicate the closed paths around singular points of the orbifold 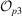 and the labels α, β indicate identified edges. See text for further details.

*Description of the orbifold structure of 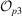 .* The space 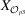 is obtained from the ASU by identifying the two edges between points *A* and *B* in Fig. 4 ▸. The result of such an identification constitutes a 1-cell denoted α. Similarly we obtain a 1-cell denoted β. The top left and bottom right corners are identified and form a 0-cell *A*. Corners *B* and *C* are not identified and they form separate 0-cells of 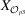 . The parallelogram *ABAC* forms the unique 2-cell of the orbifold. Topologically, the space 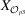 is identified with a 2D sphere. Each point 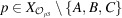 has a neighborhood *U*_*p*_ such that for an open disk 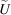 of radius 1 in 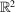 and the trivial group Γ = {0} acting on 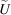 there exists a homeomorphism 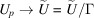 . For each point *p* ∈ {*A*, *B*, *C*} there exists a neighborhood of that point *U*_*p*_ which is homeomorphic with the unit open disk 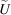 of radius 1 divided by the group action of Γ = *C*_3_, a cyclic group of order 3 generated by the counterclockwise rotations around the center point (0, 0) of the disk, namely there exists a homeomorphism 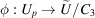 where ϕ(*p*) = (0, 0). A circle of radius 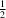 centered at (0, 0) in 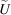 is mapped through ϕ to the corresponding image which we depict in Fig. 4 ▸. For *p* = *A*, this image is the loop γ_1_ ∪ γ_4_, for *p* = *B*, the loop is γ_2_, and for *p* = *C*, the loop is γ_3_. Note that in Fig. 4 ▸ the points *x*, *y*, *w*, *z* represented on the ASU are suitably identified. The space 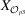 with the atlas of charts for each point 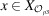 described above constitutes the structure of the orbifold 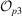 .

#### 4.1.2. The wallpaper group *p*6   

Here the ASU has a deltoidal shape, with area 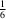 of the unit cell, and populates the plane according to sixfold symmetry, as shown in Fig. 3 ▸(*b*). The orbifold 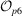 has one vertex of weight 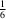 (at the sixfold axis), one of weight 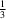 (at the threefold axis) and one of weight 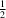 . Each of the four edges has weight 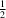 , and the deltoidal face has a weight of 1. The total vector of weighted *k*-cells is 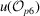 = 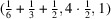 = (1, 2, 1) and 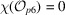 .

#### 4.1.3. The wallpaper group *p*6*mm*   

In this group the ASU is a triangle with area 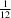 of the unit cell and populates the plane according to the *p*6*mm* planar group [Fig. 3 ▸(*c*)]. The orbifold 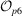 has one vertex of weight 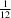 (at the point with symmetry 6*mm*), one vertex of weight 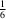 (at symmetry 3*m*) and one with weight 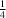 (at symmetry 2*mm*). There are three edges of weight 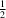 and, obviously, one interior face with a full weight of 1. The total vector in this group is 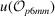 = 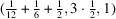 = 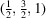 , hence 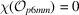 .

#### 4.1.4. The space group *Pcc*2   

**Figure 5:**
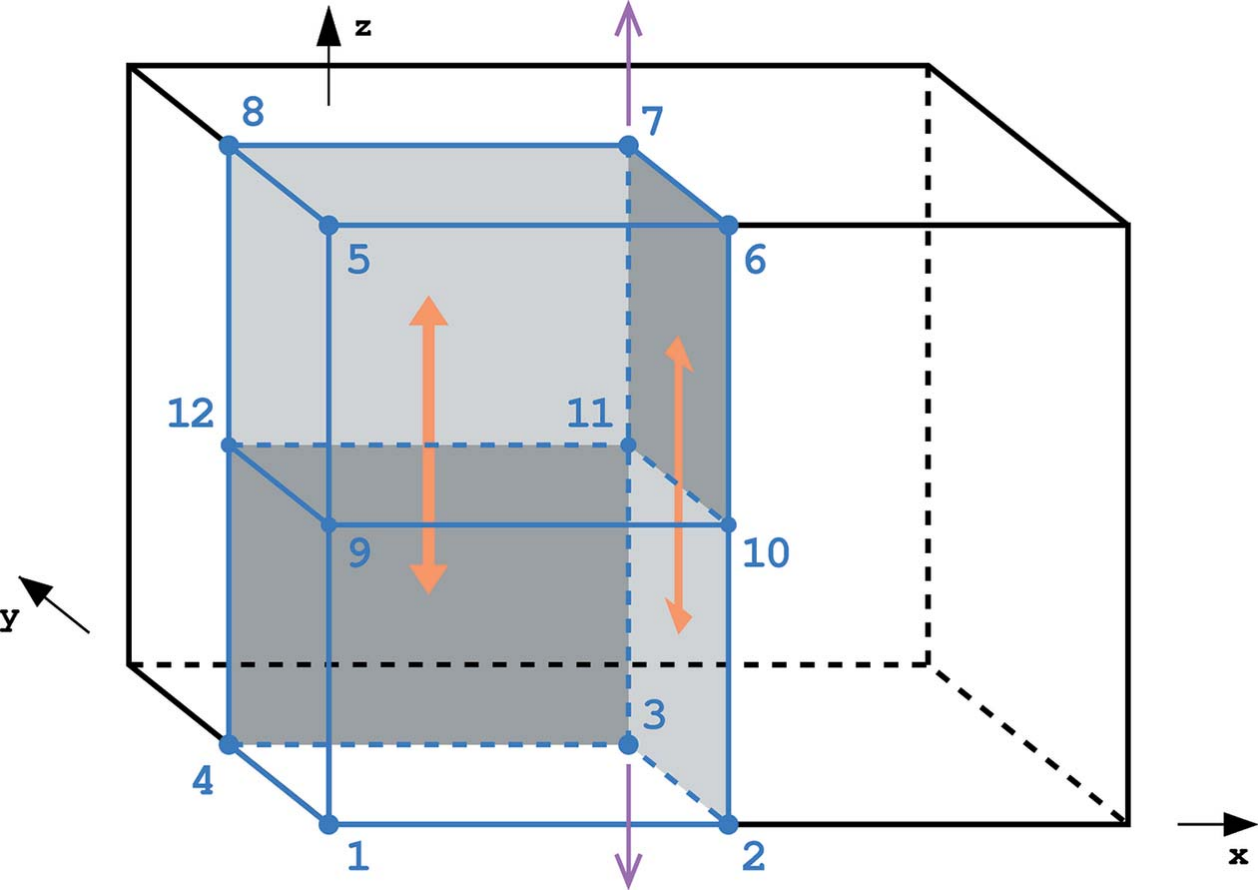
The unit cell (black edges) and ASU (blue edges) of the orthorhombic space group *Pcc*2. Only some relevant symmetry elements are marked, the twofold axis in purple and the glide *c*-planes indicated by orange arrows. Additional parallel twofold axes pass through all corners and half-edges of the cell, and additional glide planes lie at all vertical faces of the unit cell.

The symmetry relations in the space group *Pcc*2 are illustrated in Fig. 5 ▸. In this group the ASU is formed by one quadrant of the unit cell. Since the vertical faces of the ASU lie at the glide *c*-planes, these faces are divided and additional corners and edges have to be counted as building *k*-cells of this ASU. Only four corners at *z* = 0 and located on twofold axes are unique with a weight of 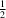 , while the other corners are symmetry equivalent by the lattice translations or glide planes. Only four edges at *z* = 0 are unique with a full weight of 1, and only the lower four vertical edges are unique with a weight of 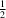 . The unique basic face at *z* = 0 has a weight of 1, and each of the four unique lower vertical faces also has a weight of 1. With the ASU interior of weight 1, the vector of *k*-cells is 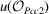 = 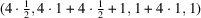 = (2, 6, 5, 1) and 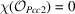 .

#### 4.1.5. The space group *P*23   

**Figure 6:**
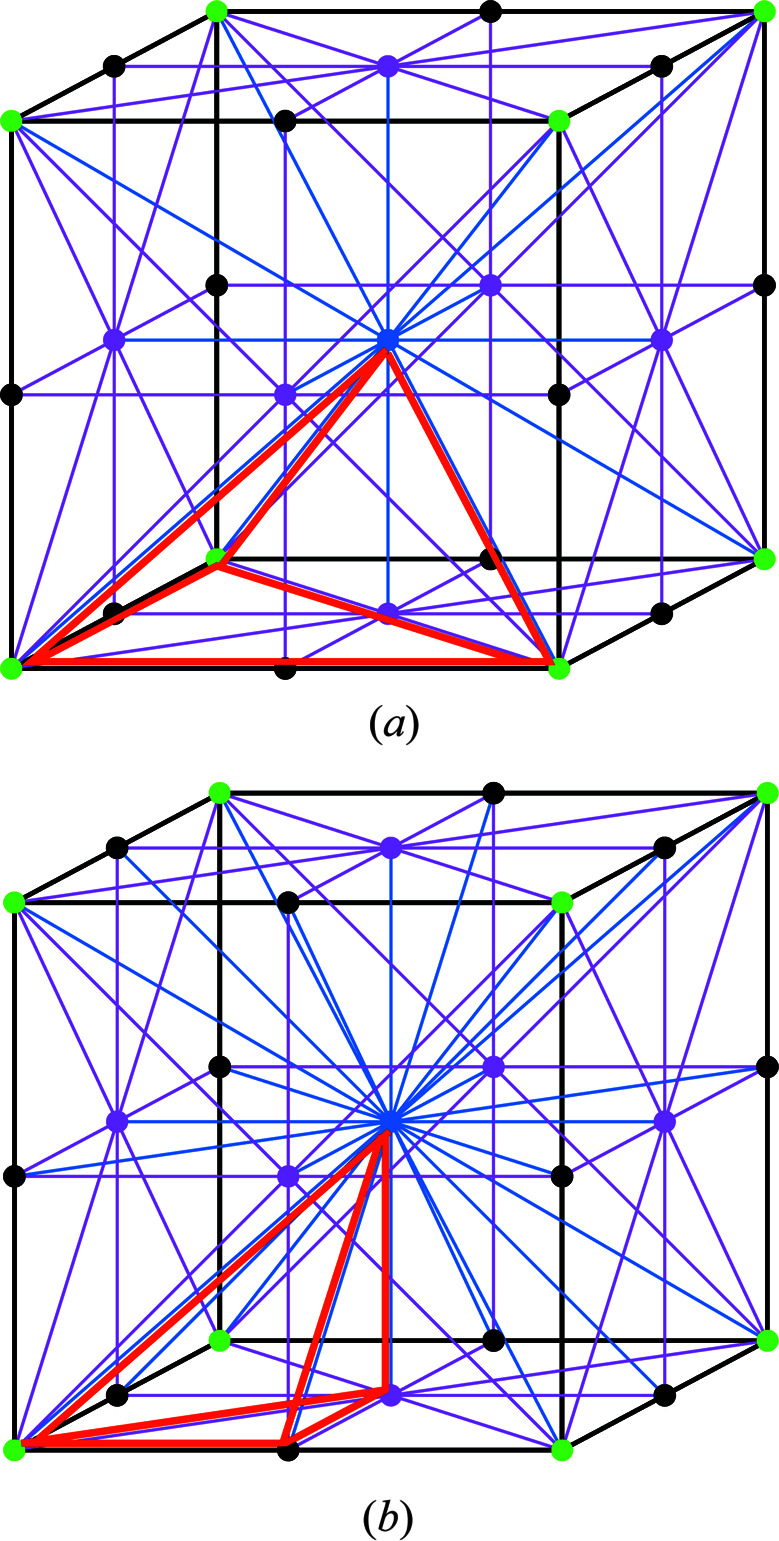
Orbifold ASU (bold red lines) of the space groups (*a*) *P*23 and (*b*) 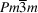 .

In this space group the counting of the contributions of weighted *k*-cells to the total vector of 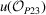 is rather complicated. Here the ASU has the shape of a tetrahedron with a volume equal to 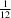 of the unit cell, but because some of its faces are bisected by the twofold axes, as illustrated in Fig. 6 ▸(*a*), it has to be counted with seven vertices, 13 edges and eight faces. Only one of the three vertices lying at the sites of 23 symmetry at three of the corners of the basic face of the cubic unit cell is unique with a weight of 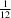 , the fourth vertex at the center of the cell has the same site symmetry and weight, one of the two vertices at the half-cell edges is unique with symmetry 222 and a weight of 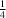 , and one vertex at the center of the basic face has the same symmetry and a weight of 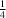 . There are three edges lying at the diagonal threefold axes having weights of 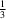 , of which only two are unique, three unique edges positioned at three perpendicular twofold axes crossing at the center of the basic face, one unique out of three diagonal edges within the basic face, and one unique out of four symmetry-equivalent edges along the full *x* and *y* edges of the basic face with a weight of 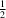 , also located on the twofold axes. All faces have a weight of 1 but only some are unique. Only one of two faces equivalent by the 23-fold axis is unique, similarly to only one of the faces equivalent by the vertical twofold axis passing through the cell center. Within the basic cell face there are two unique facets and the other two are symmetry equivalent. With the interior having a full weight of 1, the total vector in this group is 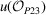 = 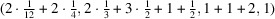 = 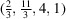 and 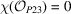 .

#### The space group 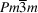


4.1.6.

The space 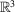 in this symmetry is populated by the ASU presented in Fig. 6 ▸(*b*), which is a tetrahedron with two vertices of weight 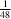 and two vertices of weight 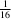 , one edge of weight 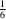 , three edges of weight 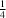 and two edges of weight 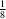 . The orbifold has four faces of weight 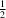 and one 3-cell of weight 1. The total vector *u* of weighted *k*-cells is (1/6, 7/6, 2, 1) and the orbifold Euler characteristic vanishes.

## 5. Coxeter’s *N_j_* summation revisited   

In this section, we reiterate the proof of Coxeter (1948 ▸) of Euler’s formula for polyhedra and its generalization to the tessellation of space with space-filling polyhedra.

Coxeter in his argument exhibited certain numbers *v*_*j*_ which are the leading terms of the growth of the number *N*_*j*_(*R*) of *j*-cells in the tessellation with respect to a given radius *R* from a fixed vertex. With a limiting argument passing from *N*_*j*_(*R*) to the limits 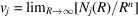 Coxeter shows that 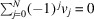 . The details of the proof are also presented in the supplementary information of the paper by Naskręcki *et al.* (2021 ▸).

This striking result should prod the attentive reader to conclude that such a formula expresses the Euler characteristic of the orbifold obtained by quotienting out the Euclidean space by the space group. This is indeed the case and we prove this relation here. In contrast to Coxeter’s approach, our proof makes no restriction on the transitivity of the vertices.

Let Γ be a crystallographic space group. Let 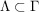 be the unique maximal normal subgroup of translations. Let Σ be a cellular decomposition of 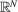 corresponding to Λ. Decomposition Σ consists of translational copies of a single parallelepiped *P*. Suppose that *U* is a polytope which, when repeated by the group Γ/Λ, imposes a further cellular decomposition of *P*. The corresponding refined cellular decomposition of 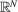 is denoted by Σ′. Let Σ′′ be a further subdivision of Σ′ such that each *k*-cell of Σ′′ has a constant stabilizer group along the interior of the *k*-cell under the action of Γ.

Theorem 5.1 Let 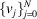 be the Coxeter parameters of Σ′′. Let μ_*j*_ denote the sum 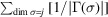 where the summation goes over the part of the subdivision Σ′′ corresponding to the polytope *U*. We prove that 

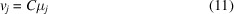

for every *j* and some explicit absolute constant *C*. In particular, since 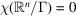 , then 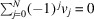 .

Proof of Theorem 5.1 Let *j* be fixed. We consider the number *N*_*j*_(*R*) of *j*-cells in the cell division Σ′′ which intersect non-trivially with a ball 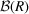 of radius *R* centered at θ = (0, …, 0) of the space. We assume that θ is a vertex of our cellular division Σ′′. Let *m*(*R*) denote the number of copies of the parallelepipeds *P* which belong to the division Σ and are completely contained in the ball 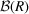 . For each translational copy λ(*P*) of *P* under λ ∈ Λ the number of translational copies of a *j*-cell σ which are contained in *P* up to translation under Λ is 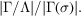 This follows from the fact that the quotient group Γ/Λ populates *P* with the copies of σ. The stabilizer group order |Γ(σ)| is the number of fixed elements which do not move σ. Hence we have the formula 

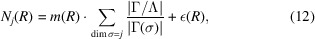

where ε(*R*) is an error function that satisfies 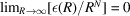 and the summation goes over the unique cells σ of dimension *j* in the orbifold 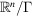 .

This leads to the conclusion that 

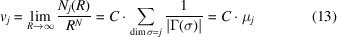

and 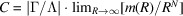 . The limit in the latter expression exists and can be computed explicitly when the explicit decomposition Σ is given. To conclude our proof we apply Theorem 4.2[Statement theorem4.2].

□

Now, we present a variant of Theorem 5.1[Statement theorem5.1] which works nicely for invariant tessellations.

Theorem 5.2 Let Σ be a cellular decomposition of 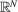 and let Γ be a crystallographic space group acting on 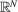 . Suppose that Σ is invariant under Γ, *i.e.* for every γ ∈ Γ and every closed cell 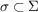 we have that γ · σ is a closed cell in Σ. Let 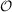 be the orbifold 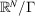 . The orbifold 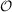 has an induced cellular decomposition from Σ. Let μ_*j*_ denote the sum 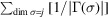 ranging over cells σ of 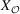 of dimension *j*.

Let 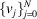 be the Coxeter parameters of Σ. Then there exists a constant *C* such that *v*_*j*_ = *C*μ_*j*_ for every *j*.

Proof of Theorem 5.2 Since Σ is invariant under Γ it follows that each closed cell σ has the stabilizer subgroup Γ_σ_ constant on the interior of σ. Let 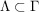 be the unique maximal normal subgroup of translations in Γ. The decomposition Σ is invariant under Λ without fixed points, hence the quotient space 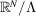 which is a torus 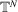 has an induced cell decomposition 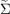 such that for each cell 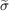 in 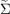 its pre-image via the projection 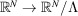 is a union of all cells λ · σ for λ ∈ Λ and for a particular lift σ of 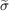 . The quotient group *G* = Γ/Λ is finite and acts on both 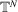 and its cell decomposition 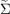 . The quotient 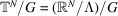 is the orbifold 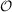 with the cellular decomposition 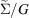 .

For each natural number *n*, let Λ_*n*_ be the subgroup of Λ (of index *n* ^*N*^) spanned by translations {*nv*}, where {*v*} is a particular set of linearly independent generators of Λ. The quotient space 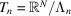 is still an *N*-dimensional torus with an induced cell decomposition 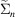 . For each cell σ in 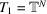 there are *n* ^*N*^ corresponding cells in *T*_*n*_. For each σ′ in 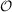 , there are |*G*|/|Γ(σ′)| pre-images in *T*_1_. Finally, we notice that the quotient of volumes of an *N*-dimensional closed ball of radius *R* and of the parallelepiped 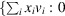 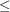 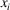 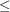 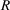 , 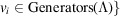 tends to a positive limit 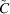 when *R* → ∞. Summing up over all cells σ′ of dimension *j* and passing to the limit *R* = *n* → ∞ proves the theorem with constant 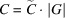 .

□

Example 5.1 Table 1 ▸ presents the formulas for functions *N*_*k*_(*n*) of variable *n* which compute the number of *k*-cells in a tessellation which respects a given group Γ, and *n* denotes the number of translation steps in each direction, starting from a fixed tessellation vertex. We note that these functions happen to be polynomials due to the periodicity of the tessellation and the choice of the counting metric. A weighted *k*-cells vector is a tuple of sums of fractions which represent the orbifold contribution of *k*-cells in each dimension taken with respect to the appropriate stabilizers. Due to the compatibility of the tessellation with the orbifold construction as in Theorem 5.1[Statement theorem5.1], the leading terms of the polynomial growth functions are in perfect agreement with the sums in the weighted *k*-cells vectors.

## 6. Applicability   

Since the present proof, in contrast to the proof by Coxeter and our own arithmetic proof, does not require the intermediate step of application to vertex transitive tessellations, it is more general and makes the modified Euler characteristic applicable to a larger class of tessellations. However, we should point out that it is still limited to tessellations which are invariant under the action of a certain crystallographic group. This is a key condition in the present proof of the vanishing of the orbifold Euler characteristic. Penrose tilings and quasicrystals violate this assumption and thus our proof is not applicable in such cases. For such tessellations different invariants exist, based on the Čech cohomology of a tiling space (Penrose, 1992 ▸).

As a final conclusion, we note that in spite of its mathematical appearance, our article is strongly rooted in crystallography. Firstly, its inspiration is fully based in the crystallographic concept of the asymmetric unit. As the classical Euler equation applies to fully bounded solids, the modified version describes crystallographic asymmetric units and Dirichlet domains. Secondly, the modified Euler characteristic χ_m_ provides a topological basis for a strictly correct (*i.e.* minimal) definition of ASU in crystallography as presented by Grosse-Kunstleve *et al.* (2011 ▸).

## Figures and Tables

**Table 1:** Polynomial *k*-cell growth functions computed for tessellations of groups *p*3, *p*6, *p*6*mm, P*23 and Pm {\overline 3} m and comparison with the orbifold weighted *k*-cell vectors *u*

*G*	*N*_*k*_(*n*)	Vector u ({\cal O}_\Gamma)	Figure
*p*3	*N*_0_(*n*) = 12*n* ^2^ + 4*n* + 1	(3 · 1/3, 2 · 1, 1)	3 ▸(*a*)
	*N*_1_(*n*) = 24*n* ^2^		
	*N*_2_(*n*) = 12*n* ^2^ − 4*n*		
*p*6	*N*_0_(*n*) = 24*n* ^2^ + 8*n* + 1	(1/6 + 1/3 + 1/2, 1 + 1, 1)	3 ▸(*b*)
	*N*_1_(*n*) = 48*n* ^2^ + 8*n*		
	*N*_2_(*n*) = 24*n* ^2^		
*p*6*m*	*N*_0_(*n*) = 24*n* ^2^ + 8*n* + 1	(1/12 + 1/6 + 1/4, 3 · 1/2, 1)	3 ▸(*c*)
	*N*_1_(*n*) = 72*n* ^2^ + 8*n*		
	*N*_2_(*n*) = 48*n* ^2^		
*P*23	*N*_0_(*n*) = 64*n* ^3^ + 48*n* ^2^ + 12*n* + 1	(2 · 1/12 + 2 · 1/4, 4 · 1/2 + 2 · 13 + 1, 4 · 1, 1)	6 ▸(*a*)
	*N*_1_(*n*) = 352*n* ^3^ + 144*n* ^2^ + 12*n*		
	*N*_2_(*n*) = 384*n* ^3^ + 96*n* ^2^		
	*N*_3_(*n*) = 96*n* ^3^		
Pm {\overline 3} m	*N*_0_(*n*) = 64*n* ^3^ + 48*n* ^2^ + 12*n* + 1	(2 · 1/48 + 2 · 1/16, 2 · 1/8 + 3 · 1/4 + 1/6, 4 · 1/2, 1)	6 ▸(*b*)
	*N*_1_(*n*) = 448*n* ^3^ + 144*n* ^2^ + 12*n*		
	*N*_2_(*n*) = 768*n* ^3^ + 96*n* ^2^		
	*N*_3_(*n*) = 384*n* ^3^		

## Appendix A Formal definitions

In this section we gather the necessary but distracting standard mathematical definitions which have been used throughout the text. For the convenience of the reader they are trimmed and adjusted to the purposes and notation of the main text.

Definition 7.1: group actions Consider a set *X* which is equipped with a (left) action of a group *G, i.e.* there is a map *m* : *G* × *X* → *X* which satisfies *m*(*g*, *m*(*h*, *x*)) = *m*(*g* · *h*, *x*) and *m*(*e*, *x*) = *x* for the identity element *e* ∈ *G*. For brevity, we write *g* · *x* instead of *m*(*g*, *x*). (i) An orbit of a point *x* ∈ *X* is *G* · *x* = {*g* · *x* : *g* ∈ *G*}. (ii) A stabilizer subgroup *G*_*x*_ of a point *x* is the subgroup {*g* ∈ *G* : *g* · *x* = *g*} of elements which fix the point *x* ∈ *X*. (iii) A quotient space *X*/*G* is the set {*G* · *x* : *x* ∈ *X*} of orbits of the action of *G* on *X*. (iv) Free action of a group *G* on *X* is an action such that *g* · *x* = *x* if and only if *g* = *e*. (v) A topological group *G* acts properly discontinuously on a topological space *X* if each point *x* ∈ *X* has an open neighborhood *U*_*x*_ such that {*g* ∈ *G* : *g* · *U*_*x*_ ∩ *U*_*x*_ ≠ ∅} is a finite set.

Let us now formalize the notion of an orbifold. We follow closely the definition of Thurston (2002 ▸; Section 13.2).

Definition 7.2 An orbifold  is a pair which constists of a Hausdorff space  and a set  of open subsets in  such that  , and for every *i* ≠ *j* if  then  . Moreover, there exists a positive integer *n* such that for each *i* ∈ *I* there exists a finite group Γ_*i*_, an open subset  , a group action of Γ_*i*_ on  and a homeo­morphism  . The maps ϕ_*i*_ satisfy the following compatibility conditions: (i) For *i*, *j* ∈ *I* if  , then there exists an injective homomorphism of groups  and an inclusion  which satisfies the condition

(ii) The following diagram of maps commutes.[Chem scheme1]

The map ϕ_*ij*_ is a natural map obtained from  by passing to the quotient by Γ_*i*_. The map η_*ij*_ is obtained from the injection *f*_*ij*_. A triple  is called a chart.

In general, the choice of two covers of the space  for an orbifold  is considered equivalent if there exists a larger (finer) cover which is built by intersections from the two (coarser) covers.

Although the definition of an orbifold looks complicated, it encodes an intuitive idea that in some quotient spaces one can find a family of compatible charts which have a finite group action.

Theorem 7.1 [Thurston (2002), Proposition 13.2.1] Let *M* be a manifold and Γ a group which is acting properly discontinuously on *M*. The quotient space *M*/Γ has the structure of an orbifold in the sense of Definition 7.2[Statement definition7.2].

A critical idea that we exploit in this article is the existence of certain covering maps between orbifolds. We encode the essential features in the following definition [modeled on Thurston (2002 ▸), Definition 13.2.2)].

Definition 7.3: orbifold cover Let  be a surjective continuous map of orbifolds, *i.e.* a surjective map of the underlying topological spaces  . Such a map *p* is a degree *d* cover of orbifolds if for each  there exists a chart  over the point  such that for each component *V*_*i*_ of *p* ^−1^(*U*) we have: (i) Each *V*_*i*_ has an orbifold chart  where Γ_*i*_ < Γ,  is a homeomorphism and *p* lifts to the identity map  . (ii) The cardinality of the pre-image set *p* ^−1^(*x*) equals *d* for any regular point, *i.e.* *G*_*x*_ = {*e*}.
